# Efficacy of Standard and Intensive Statin Treatment for the Secondary Prevention of Cardiovascular and Cerebrovascular Events in Diabetes Patients: A Meta-Analysis

**DOI:** 10.1371/journal.pone.0111247

**Published:** 2014-11-05

**Authors:** Folgerdiena M. de Vries, Johan Kolthof, Maarten J. Postma, Petra Denig, Eelko Hak

**Affiliations:** 1 University Groningen, Department of Pharmacy, Unit of PharmacoEpidemiology & PharmacoEconomics, Groningen, the Netherlands; 2 Department of Clinical Pharmacy and Pharmacology, University of Groningen, University Medical Center Groningen, Groningen, The Netherlands; Washington Hospital Center, United States of America

## Abstract

**Aims:**

To estimate the efficacy of standard and intensive statin treatment in the secondary prevention of major cardiovascular and cerebrovascular events in diabetes patients.

**Methods:**

A systematic search was conducted in Medline over the years 1990 to September 2013. Randomized, double-blind, clinical trials comparing a standard-dose statin with placebo or a standard-dose statin with an intensive-dose statin for the secondary prevention of cardiovascular and cerebrovascular events in diabetes patients were selected. Trial and patient characteristics were extracted independently by two researchers. The combined effect on the composite primary endpoint was measured with a fixed-effect model. Potential publication bias was examined with a funnel plot.

**Results:**

Five trials were included in the analysis comparing standard-dose statins with placebo with a total of 4 351 participants. Four trials were included for comparing standard-dose with intensive-dose statins, including 4 805 participants. Compared with placebo, standard-dose statin treatment resulted in a significant relative risk (RR) reduction of 15% in the occurrence of any major cardiovascular or cerebrovascular event (RR 0.85, 95% CI 0.79–0.91). Compared with standard-dose statin treatment, intensive-dose statin treatment resulted in an additional 9% relative risk reduction (RR 0.91, 95% CI 0.84–0.98).

**Conclusion:**

Treatment with standard-dose statins to prevent cardiovascular or cerebrovascular events in diabetes patients with manifest cardiovascular disease results in an estimated 15% relative risk reduction and intensive-dose statin treatment adds 9%. If proven cost-effective, more intensive statin treatment should be recommended for diabetes patients at high cardiovascular risk.

## Background

Cardio- and cerebrovascular diseases are ranked among the major causes of mortality worldwide [1]. Patients with diabetes have a two- to four-fold higher risk of cardiovascular events than age-matched non-diabetes patients [2]. Especially those diabetes patients with a history of cardio- or cerebrovascular disease are at increased risk for recurrent events [3]. Hence, cardiovascular risk management is an essential part of the management of diabetes [4].

The need for statin treatment for secondary prevention of cardiovascular events is widely recognized. Significant benefits of statin treatment were reported in two meta-analyses, leading to the conclusion that statin therapy reduces the occurrence of major vascular events in diabetes patients with and without vascular disease [5], [6]. These analyses did not include the ASPEN study, which did not show significant results with a standard-dose statin in diabetes patients [7]. Recently Chang et al. [8] performed a meta-analysis and came to the conclusion that there is still uncertainty regarding the benefits of statins in diabetes patients. When focusing on high quality trials for secondary prevention in diabetic patients only, being the ASPEN trial and the 4D trial [9], no significant benefits of statin treatment were seen [8]. In these previous meta-analyses several studies with selective subgroups, such as patients on hemodialysis, were included and a significant heterogeneity was seen. Moreover, most trials included in the previous meta-analyses were limited to interventions with standard-dose statins. Baigent et al. reported, however, that more intensive treatment is associated with even further reductions in the risk for major vascular events in secondary prevention patients [10]. As LDL cholesterol targets have been set at lower levels, targets are not met in at least a third of these patients and more intensive treatment may be needed [11], [12]. Randomized clinical trials comparing standard with intensive statin treatment reported partly significant and partly insignificant effects of intensive treatment on reducing the occurrence of major cardiovascular or cerebrovascular complications within the diabetes subgroup [13], [14], [15], [16]. For clinical decision making and cost-effectiveness analysis it is important to have precise effect estimates of standard and intensive statin treatment for secondary prevention diabetes patients.

We aimed to perform two meta-analyses: (1) to assess the effect of standard-dose statins over placebo and (2) to assess the additional effect of intensive-dose statins over standard-dose statins, both for the secondary prevention of major cardiovascular and cerebrovascular events in a non-restrictive diabetes population. Only high-quality and double-blinded studies were eligible for inclusion.

## Methods

### Search strategy

We searched the Medline and Embase databases and ClinicalTrials.gov for randomized clinical trials that either compared statin treatment to placebo or standard to intensive statin treatment for the secondary prevention of cardiovascular and cerebrovascular events (1990 to September 2013) reported in the English-language. Trials were identified with the use of the medical subject heading (MeSH) terms ‘statins’, ‘HMG-CoA reductase inhibitor’, ‘randomized controlled trial’, ‘secondary prevention’, ‘intensive’, ‘moderate’, ‘diabetes’, ‘coronary heart disease’, ‘myocardial infarction’ and ‘stroke’. Reference lists of reviews and meta-analysis related to the study were examined. The search was independently performed by two researchers (FV and JK).

### Study selection

Inclusion criteria for both meta-analyses were: randomized clinical trials (1); including a non-restrictive secondary prevention diabetes population with previous coronary heart disease, cerebrovascular disease, myocardial infarction or unstable angina pectoris (2); and reporting major cardiovascular and cerebrovascular events as endpoint (3). For the first meta-analysis studies were required to include a standard-dose statin arm and a placebo arm, for the second meta-analysis studies were included which compared standard-dose with intensive-dose statin arm. Standard-dose refers to commonly prescribed daily doses of atorvastatin < = 20 mg, simvastatin < = 60 mg, rosuvastatin < = 10 mg and any dosing of pravastatin, lovastatin and fluvastatin [17], [18], [19]. Higher daily doses were categorized as intensive-dose statin treatment. The quality of selected studies was scored with the Jadad score [20]. The Jadad score evaluates on a scale from 0–5 the appropriateness of the randomization technique (1), the method for double-blinding (2) and the description of withdrawals and dropouts (3). Two researchers (JK, FV) individually extracted the trial and patient characteristics and the outcome results.

### Endpoints

The primary endpoint for both meta-analyses was a composite of major cardiovascular and cerebrovascular events, including the first occurrence of fatal and non-fatal myocardial infarction (MI), fatal and non-fatal stroke, revascularization and hospitalization for unstable angina. The meta-analysis comparing standard-dose statins with placebo also assessed the secondary endpoints: fatal and non-fatal MI (a); fatal and non-fatal stroke (b); and all-cause mortality (c).

### Data analysis and statistical methods

For each endpoint, the relative risk (RR) with corresponding 95% confidence interval (CI) and the number needed to treat (NNT) with 95% CI were calculated [21]. The results of the separate studies were pooled with the fixed-effect model and the random-effect model in the computer program RevMan from the Cochrane Collaboration [22], [23]. The fixed-effect model assumes that differences in effects between studies are due to sampling error whereas the random-effect model assumes that the separate studies are measuring different effects. Model selection was based on heterogeneity testing which was assessed by calculating the Q statistic, that tests the homogeneity hypothesis, and the I^2^ index [24], that calculates the percentage of variability in the effect estimates that is due to heterogeneity rather than chance. Based on the heterogeneity testing the fixed-effect model was used for all endpoints. Evidence for potential publication bias was examined by visually studying funnel plots.

## Results

### Description of included randomized clinical trials

**Figure 1:**
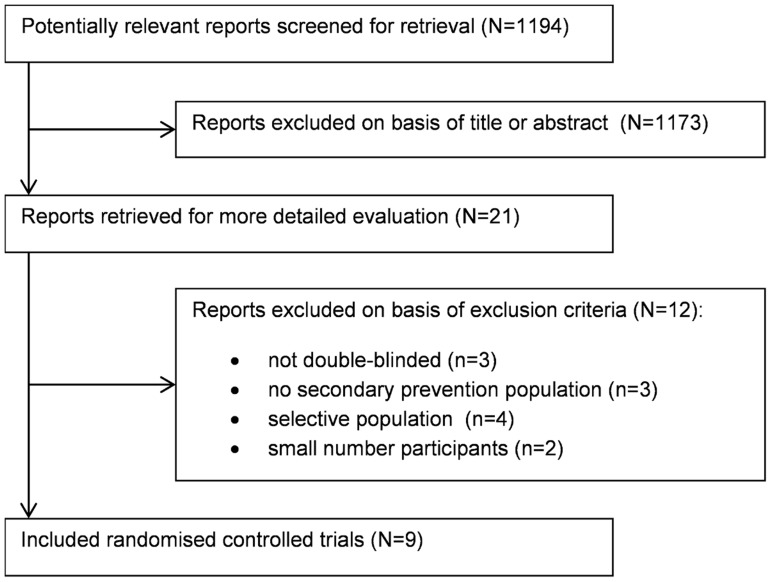
Flow diagram of study selection.

Data from nine randomized clinical trials were included in the current meta-analyses (Fig. 1): the 4S [25], the ASPEN [7], the CARE [26], the HPS [27] and the LIPID [28] trial for the comparison of a standard-dose statin with placebo and the A to Z [13], the PROVE-IT TIMI [14], the SEARCH [15] and the TNT [16] trials for the comparison of standard-dose statin treatment with intensive-dose statin treatment (see Table 1 for full trial names). The study and patient characteristics of the included studies can be found in Table 2 and Table 3. In total, 4 351 (2 153 standard-dose statin/2 198 placebo) participants were included in the standard-dose statin/placebo analysis and 4 805 (2 409 intensive-dose statin/2 396 standard-dose statin) participants were included for the comparison intensive-dose statin/standard-dose statin treatment. Within the standard-dose statin/placebo analysis participants were treated with pravastatin 40 mg, atorvastatin 10 mg or simvastatin in the dosage of 20 or 40 mg daily. Within the analysis comparing standard-dose statin treatment with intensive-dose statin treatment, patients in the standard-dose statin group were treated with simvastatin 20 mg, pravastatin 40 mg or atorvastatin 10 mg and patients in the intensive-dose statin group were treated with simvastatin 80 mg or atorvastatin 80 mg.

**Table 1:** Full trial name of study acronyms.

Study acronyms	Trial name
4D [9]	Die Deutsche Diabetes Dialyse Studie
4S [25]	Scandinavian Simvastatin Survival Study
ASPEN [7]	Atorvastatin Study of Prevention of coronary heart disease Endpoints in Non-insulin-dependent diabetes mellitus
A to Z [13]	Phase Z of the A to Z trial
CARE [26]	Cholesterol And Recurrent Events
GISSI [30]	Gruppo Italiano per lo Studio della Sopravvivenza nell'Infarto Miocardico Prevenzione trial
HPS [27]	Heart Protection Study of cholesterol-lowering with simvastatin in people with diabetes
LIPID [28]	Long-Term Intervention with Pravastatin in Ischemic Disease
LIPS [31]	The Lescol Intervention Prevention Study
Post CABG [29]	Post Coronary Artery Bypass Graft Trial
PROVE IT TIMI [14]	The Pravastatin or Atorvastatin Evaluation and Infection Therapy Thrombolysis in Myocardial Infarction trial
SEARCH [15]	Study of the Effectiveness of Additional Reductions in Cholesterol and Homocysteine
TNT [16]	Treating to New Targets

**Table 2:** Patient and trial characteristics of the included studies.

Trial	Intervention	Patients	Drugs	DM type	Outcome used for primary endpoint	Jadad [20] #
4S,^1997^ [25]	SDS/Placebo	MI or AP	Sim 20 mg	T1/T2	CHD death, MI, revascularization, stroke, PVE	5
ASPEN,^2006^ [7]	SDS/Placebo	MI or IP	Ato 10 mg	T2	CHD death, MI, stroke, revascularization, UAP	4*
CARE,^1998^ [26]	SDS/Placebo	MI	Pra 40 mg	T1/T2	CHD death, MI, revascularization	5
HPS,^2003^ [27]	SDS/Placebo	CVD	Sim 40 mg	T1/T2	CHD death, MI, stroke, revascularization	5
LIPID,^2003^ [28]	SDS/Placebo	MI or UAP	Pra 40 mg	T1/T2	CHD death, MI, stroke, revascularization, UAP	5
A to Z,^2004^ [13]	SDS/IDS	ACS	Sim 20 mg/Sim 80 mg	T1/T2	CHD death, MI, stroke, AP	5
PROVE-IT TIMI,^2006^ [14]	SDS/IDS	ACS	Pra 40 mg/Ato 80 mg	T1/T2	CHD death, MI, stroke, AP, revascularization	4*
SEARCH,^2010^ [15]	SDS/IDS	MI	Sim 20 mg/Sim 80 mg	T1/T2	CHD death, MI, stroke, revascularization	5
TNT,^2006^ [16]	SDS/IDS	CHD	Ato 10 mg/Ato 80 mg	T1/T2	CHD death, MI, stroke, revascularization, AP, CHF	4*

# score ranging 1–5; * method of randomization not described.

**ACS:** acute coronary syndrome; **AP:** angina pectoris; **Ato:** atorvastatin; **CHD:** coronary heart disease; **CVD:** cardiovascular disease; **CHF:** congestive heart failure; **DM:** diabetes mellitus; **IDS:** intensive-dose statin; **IP:** interventional procedures; **LDLC:** low-density lipoprotein cholesterol; **MI:** myocardial infarction; **Pra:** pravastatin; **PVE:** peripheral vascular event; **SDS:** standard-dose statin; **Sim:** simvastatin; **T1:** Type 1 diabetes; **T2:** Type 2 diabetes; **UAP:** unstable angina pectoris.

**Table 3:** Patient characteristics of the included studies.

Trial	No. DM patients (exp/con)	Age (yr.)	Men (%)	Baseline TC (mmol/l)	Baseline LDLC (mmol/l)	Follow-up (yr.)
4S,^1997^ [25]	202 (105/97)	60	72	6.7	4.8	5.4
ASPEN,^2006^ [7]	505 (252/253)	63	82	4.9	2.9	4.0
CARE,^1998^ [26]	586 (282/304)	61	80	5.3	3.6	5.0
HPS,^2003^ [27]	1981 (972/1009)	NA	NA	NA	NA	5.0
LIPID,^2003^ [28]	1077 (542/535)	64	81	5.6	3.7	6.0
A to Z,^2004^ [13]	1059 (529/530)	NA	NA	NA	NA	2
PROVE-IT TIMI,^2006^ [14]	978 (499/479)	60	72	4.6	2.6	2
SEARCH,^2010^ [15]	1267 (633/634)	NA	NA	NA	NA	6.7
TNT,^2006^ [16]	1501 (748/753)	63	72	4.5	2.5	5

**Con:** controls; **DM:** diabetes mellitus; **Exp:** experimental; **LDLC:** low-density lipoprotein cholesterol; **TC:** total cholesterol.

The weighted mean follow-up was 5.3 years (ranging from 4.0 in the ASPEN trial [7] and 6.0 in the LIPID trial [28]) in the standard-dose statin/placebo analysis and 4.6 years (ranging from 2.0 years in the A to Z trial [13] and the PROVE-IT TIMI trial [14] to 6.7 years in the SEARCH trial [15]) in the standard-dose statin/intensive-dose statin analysis. Participants in both analyses did not differ according to age and gender.

The definition for secondary prevention patients was slightly different for the individual trials included in both meta-analyses (Table 2). Some studies included patients with previous unstable angina or cerebrovascular disease, while others did not. Furthermore, some slight differences in inclusion criteria were made in age, cholesterol and triglyceride levels. Small differences were also present in the definition of endpoints that were included in the composite endpoint of major cardiovascular and cerebrovascular events.

### Relative risk of standard-dose statin versus placebo

**Figure 2:**
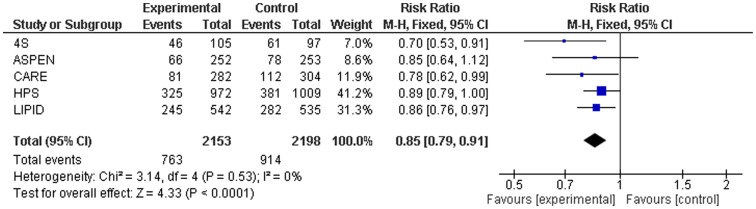
Results of the primary endpoint of major cardiovascular and cerebrovascular events comparing standard-dose statins with placebo.

**Figure 3:**
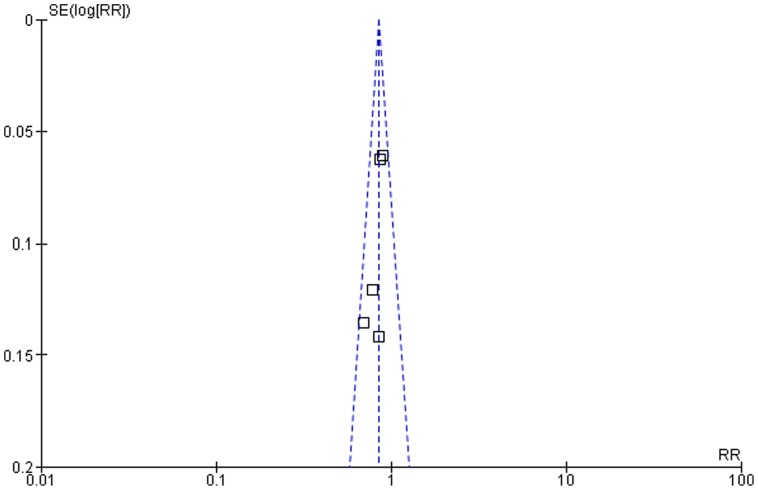
Funnel plot of the meta-analysis comparing placebo with standard-dose statin treatment.

Within a hypothetical high risk population with a 10-year risk for cardiovascular disease of 50%, 27 patients need to be treated for 5 years with a standard-dose statin to prevent one major cardiovascular or cerebrovascular event. Standard-dose statin treatment was associated with a significant relative risk (RR) reduction of 15% for major cardiovascular or cerebrovascular events (RR 0.85, 95% CI 0.79–0.91) (Fig. 2, Fig. 3). In the statin treatment group, 35.2% of subjects had a major cardiovascular or cerebrovascular event (763/2153), whereas 41.4% of those in the placebo group experienced such an event (914/2198). There was no observed publication bias and non-significant heterogeneity in the individual effect estimates.

In Table 4, participant and event numbers and outcome results for secondary endpoints are presented. For fatal and non-fatal stroke, there was a significant 33% relative risk reduction (RR 0.67, 95% CI 0.49–0.90). Non-significant relative risk reductions of 27% for fatal and non-fatal MI (RR 0.73, 95% CI 0.53–1.00) and 22% for all-cause mortality (RR 0.78, 95% CI 0.53–1.14) were found.

**Table 4:** Overall results of the primary and secondary endpoints in the meta-analysis comparing standard-dose statins with placebo and intensive-dose statins with standard-dose statins.

	No. of patients(statin/control)	No. of events(statin/control)	RR (95% CI)	I^2^	Q	NNT (95% CI)
**Standard-dose vs. placebo:**						
MCCE	2153/2198	763/914	0.85 (0.79–0.91)	0%	3.14	16 (11–30)
F/NF stroke	1181/1189	65/98	0.67 (0.49–0.90)	0%	2.0	37 (21–142)
F/NF MI	534/557	56/81	0.73 (0.53–1.0)	0%	0.22	25 (13–711)
All-cause mortality	357/350	41/51	0.78 (0.53–1.14)	41%	1.70	32 (12–∞)
**Intensive-dose vs. standard-dose:**						
MCCE	2409/2396	764/837	0.91(0.84–0.98)	0%	0.04	31 (17–180)

**F:** fatal; **MCCE:** Major cardiovascular and cerebrovascular events; **MI:** myocardial infarction; **NF:** non-fatal.

### Relative risk of intensive-dose statin versus standard-dose statin

**Figure 4:**
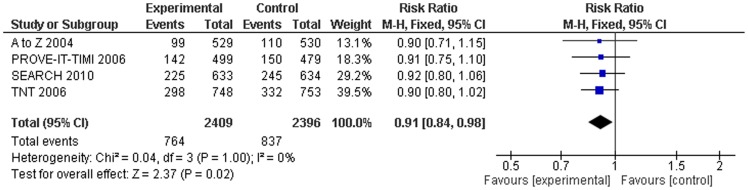
Results of the primary end point of major cardiovascular and cerebrovascular events comparing standard-dose statins with intensive-dose statins.

**Figure 5:**
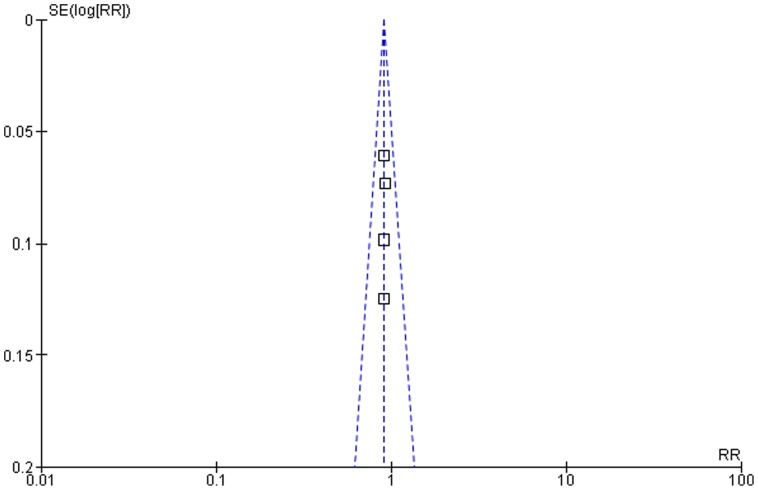
Funnel plot of the meta-analysis comparing standard-dose with intensive-dose statin treatment.

To prevent one major cardiovascular or cerebrovascular event, 17 patients need to be treated with an intensive-dose statin for 5 years in a hypothetical high risk population with a 10-year risk for cardiovascular disease of 50%. Compared to treatment with standard-dose statins, treatment with intensive-dose statins resulted in an additional 9% relative risk reduction (RR 0.91, 95% CI 0.84–0.98) (Fig. 4, Fig. 5, Table 4). In the intensive-dose statin treatment group, 31.7% of subjects had a major cardiovascular or cerebrovascular event (764/2409), whereas 34.9% of those in the standard-dose statin treatment group experienced such an event (837/2396).

## Discussion

The results of this study show that treatment with standard-dose statins for the secondary prevention of major cardiovascular or cerebrovascular events in diabetes patients is associated with a 15% significant relative risk reduction. Treating patients with an intensive-dose statin instead of a standard-dose statin will reduce the relative risk for such events with an additional 9%. Within a hypothetical high risk population with a 10-year risk for cardiovascular events of 50% this will reduce the number needed to treat for 5 years to prevent one major cardiovascular and cerebrovascular event from 27 to 17.

Secondary endpoints in the analysis of a standard-dose statin compared to placebo also achieved a significant relative risk reduction of 33% for fatal and non-fatal stroke and non-significant relative risk reductions of 27% for fatal and non-fatal MI and 22% for all-cause mortality. The number of participants for these analyses were small, however, leading to wide confidence intervals. Due to lack of data we were not able to compare standard-dose statin treatment and intensive-dose statin treatment for secondary endpoints in a meta-analysis. The TNT [16] and PROVE-IT TIMI [14] trials, however, did report results on some of these endpoints. In the TNT trial there was a non-significant risk reduction of 33% (RR 0.67, 95% CI 0.43–1.04) for stroke. The PROVE-IT TIMI trial reported a non-significant difference in stroke event rate of 2.6% for diabetes patients on intensive-dose statin treatment vs 2.2% on standard-dose statin treatment. Also, regarding event rates for MI and all-cause mortality, only non-significant differences were observed [14], [16].

Previous meta-analyses comparing standard-dose statin treatment with placebo for secondary prevention in diabetes patients reported risk reductions of 20% [5] and 21% [6] as well as a non-significant reduction of 11% [8], whereas our estimate was 15%. These differences can be explained by differences in study inclusion criteria. The estimates of 20% and 21% included non-blinded studies, such as the Post-CABG [29] in the analysis of Costa et al. [6] and the GISSI [30] in the analysis of Kearney et al. [5]. Furthermore, Chang et al. included studies in restrictive subpopulations, such as patients with heart failure or on hemodialysis [8]. Analyses of Chang et al. showed that including these studies resulted in higher risk reductions than when including only double-blinded studies in diabetes patients [8]. All previous analyses included the LIPS [31] which was conducted in patients after a successful percutaneous coronary intervention. We did not include non-blinded studies nor studies in restrictive subpopulations.

On the other hand, we did include the ASPEN trial that was not included by Kearney et al. [5] and Costa et al. [6]. The non-significant results from the ASPEN trial caused conflicting evidence from the randomized clinical trials regarding the efficacy of statins [7] but contributed to our risk reduction estimate of 15%.

In addition to the previous meta-analyses comparing standard-dose statins with placebo, we conducted a meta-analysis comparing standard-dose with intensive-dose statins including all diabetes patients from relevant studies. Through using similar inclusion criteria for both comparisons, we were able to determine the estimate of the overall effect of statins in a general secondary prevention population with diabetes and the effect of intensive-dose over standard-dose statins.

There are some limitations to our study. There were small differences in the definition for secondary prevention in the separate studies, which could have resulted in differences in the baseline risk of the populations. Also, the diagnostic criteria for diabetes differed among the studies. Where most used the World Health Organization definition for diabetes, CARE [26] interviewed the patients and asked whether they had diabetes. Most included type 1 and type 2 diabetes patients. Furthermore, the events included in the primary endpoint were not exactly similar. The differences especially concern the inclusion of unstable angina, congestive heart failure or peripheral vascular events in addition to CHD death, MI and stroke in some studies. Including additional events in a composite endpoint may lead to larger or smaller risk reductions depending on the effect of statins on such events. The larger risk reductions seen for our secondary endpoints MI and stroke may suggest that we have underestimated the overall risk reduction for the major events. Furthermore, not all patients who were prescribed statins were still taking them at the end of follow-up, and some patients in the placebo group may also receive statins during follow-up, which will lead to lower risk reductions. In the analysis comparing standard-dose with intensive-dose statin treatment, patients could also have changed treatment intensity over time. For the ASPEN [7], HPS [27], TNT [16], PROVE-IT TIMI [14], A to Z [13] and SEARCH [15] trials, we used subgroup results which can cause bias. However, these trials used minimization randomization techniques we do not expect that this will have a high impact on the meta-analysis results.

Cardiovascular risk management is an important part of diabetes treatment. The Dutch and European guidelines [32], [33], for example, recommend statin treatment in almost all diabetes patients, and especially in those patients with a history of cardiovascular disease. LDL-cholesterol targets are lowered for these high risk patients, therefore reaching targets is more difficult and intensive-dose statin treatment may be needed [11], [12], [34]. Our analysis shows that intensive-dose statin treatment has a significant effect in reducing major cardiovascular and cerebrovascular events compared to standard-dose statin treatment for the secondary prevention in diabetes patients. We should acknowledge, however, that these reductions are not confirmed yet by analyzing individual endpoints. While the 2012 ESC guidelines just recommend statin treatment [33], the recent ACC/AHA guideline recommends intensive-dose statin treatment for all secondary prevention patients [35]. In contrast, the current Dutch guidelines recommend to start with a standard-dose statin, such as simvastatin 40 mg. This choice is largely driven by economic considerations [32]. Now that the patent for atorvastatin has expired, however, the cost-effectiveness needs to be reassessed.

Besides the reductions in cardiovascular events, there are adverse events associated with statins, among which muscle toxicity and effects on liver enzymes are well acknowledged [36]. With higher doses there is an increased risk for statin-induced adverse events, especially regarding their effect on liver enzymes [36], [37], [38]. The risk of myopathy remains low at high doses, with an estimated incidence of 0,5% for simvastatin 80 mg and 1.5% for atorvastatin 80 mg [37], [38]. The incidence of rhabdomyolysis was found to be too low to detect significant differences between standard and intensive dose statin treatment [36], [37], [38].

The efficacy of statins as shown here is based on clinical trials in which patients usually form a more selective population than patients included in observational studies. A few observational studies have been conducted in secondary prevention patients. These studies show similar results as found in the clinical trials. Significant risk reductions for repeat hospitalization for acute coronary syndrome and for mortality were reported in observational studies comparing standard-dose with intensive-dose statins [39], [40].

Translation of trial evidence into guideline recommendations and of guideline recommendations into practice is subject to interpretation of the evidence. Meta-analyses can support and strengthen this process. Some guidelines now recommend intensive-dose statin treatment for all secondary prevention patients, whereas others recommend to start statin treatment on a standard-dose and to switch to an intensive-dose if LDL cholesterol targets are not reached. Our meta-analyses show that standard-dose statin treatment is associated with a 15% relative risk reduction of cardiovascular and cerebrovascular events and that the use of intensive-dose statins results in a reduction of 9% compared to standard-dose statin treatment in a secondary prevention diabetes population. These estimates are useful for further cost-effectiveness analyses. If proven cost-effective, a more differentiated advise can be given, where more intensive treatment is recommended for diabetes patients at high cardiovascular risk.

## Supporting Information

Checklist S1 **PRISMA 2009 checklist.**
(DOC)

## References

[1] World Health organisation (2012) Global Health Observatory 2012. Available: http://www.who.int/gho/mortality_burden_disease/en/. Accessed 2014 Sept 8.

[2] American Heart Association (2013) Cardiovascular disease and diabetes [online]. Available: http://www.heart.org/HEARTORG/Conditions/Diabetes/WhyDiabetesMatters/Cardiovascular-Disease-Diabetes_UCM_313865_Article.jsp. Accessed 2014 Sept 8.

[3] National Cholesterol Education Program (NCEP) Expert Panel on Detection, Evaluation, and Treatment of High Blood Cholesterol in Adults (Adult Treatment Panel III) (2002) Third Report of the National Cholesterol Education Program (NCEP) Expert Panel on Detection, Evaluation, and Treatment of High Blood Cholesterol in Adults (Adult Treatment Panel III) final report. Circulation 106: 3143. 12485966

[4] Buse JB, Ginsberg HN, Bakris GL, Clark NG, Costa F, et al (2007) Primary prevention of cardiovascular diseases in people with diabetes mellitus: a scientific statement from the American Heart Association and the American Diabetes Association. Diabetes Care 30: 162–172. 17192355 10.2337/dc07-9917

[5] Kearney PM, Blackwell L, Collins R, Keech A, Simes J, et al (2008) Efficacy of cholesterol-lowering therapy in 18 686 people with diabetes in 14 randomised trials of statins: a meta-analysis. Lancet 371: 117–125. 18191683 10.1016/S0140-6736(08)60104-X

[6] Costa J, Borges M, David C, Vaz Carneiro A (2006) Efficacy of lipid lowering drug treatment for diabetic and non-diabetic patients: meta-analysis of randomised controlled trials. BMJ 332: 1115–1124. 16585050 10.1136/bmj.38793.468449.AE PMC1459619

[7] Knopp RH, D’Emden M, Smilde JG, Pocock SJ (2006) Efficacy and safety of atorvastatin in the prevention of cardiovascular end points in subjects with type 2 diabetes: the Atorvastatin Study for Prevention of Coronary Heart Disease Endpoints in non-insulin-dependent diabetes mellitus (ASPEN). Diabetes Care 29: 1478–1485. 16801565 10.2337/dc05-2415

[8] Chang YH, Hsieh MC, Wang CY, Lin KC, Lee YJ (2013) Reassessing the benefits of statins in the prevention of cardiovascular disease in diabetic patients - A systematic review and meta-analysis. Rev Diabet Stud 10: 157–170. 24380090 10.1900/RDS.2013.10.157 PMC4063097

[9] Warner C, Krane V, Marz W, Olschewski M, Mann JF, et al (2005) Dialysis Study I. Atorvastatin in patients with type 2 diabetes mellitus undergoing hemodialysis. N Engl J Med 353: 238–248. 16034009 10.1056/NEJMoa043545

[10] Baigent C, Blackwell L, Emberson J, Holland LE, Reith C, et al (2010) Efficacy and safety of more intensive lowering of LDL cholesterol: a meta-analysis of data from 170 000 participants in 26 randomised trials. Lancet 376: 1670–1681. 21067804 10.1016/S0140-6736(10)61350-5 PMC2988224

[11] Heintjes EM, Penning-van Beest FJA, Plat AW, Meerding WJ, Webb K, et al (2012) Cholesterol level goal attainment with statins: clinical management guideline recommendations versus management in actual clinical practice. Pharmacotherapy 32: 631–641. 22760692 10.1002/j.1875-9114.2011.01086.x

[12] Sidorenkov G, Haaijer-Ruskamp FM, de Zeeuw D, Denig P (2011) A longitudinal study examining adherence to guidelines in diabetes care according to different definitions of adequacy and timeliness. PLoS One 6: e24278. 21931669 10.1371/journal.pone.0024278 PMC3169586

[13] De Lemos JA, Blazing MA, Wiviott SD, Lewis EF, Fox KA, et al (2004) Early intensive vs a delayed conservative simvastatin strategy in patients with acute coronary syndromes. Phase Z of the A to Z trial. JAMA 292: 1307–1316. 15337732 10.1001/jama.292.11.1307

[14] Ahmed S, Cannon CP, Murphy SA, Braunwald E (2006) Acute coronary syndromes and diabetes: is intensive lipid lowering beneficial? Results of the PROVE IT-TIMI 22 trial. European Heart Journal 27: 2323–2329. 16954134 10.1093/eurheartj/ehl220

[15] Armitage J, Bowman L, Wallendszus K, Bulbulia R, Rahimi K, et al (2010) Intensive lowering of LDL cholesterol with 80 mg versus 20 mg simvastatin daily in 12 064 survivors of myocardial infarction: a double-blind randomised trial. Lancet 376: 1658–1669. 21067805 10.1016/S0140-6736(10)60310-8 PMC2988223

[16] Shepherd J, Barter P, Carmena R, Deedwania P, Fruchart J, et al (2006) Effect of lowering LDL cholesterol substantially below currently recommended levels in patients with coronary heart disease and diabetes. The treating to new targets (TNT) study. Diabetes Care 29: 1220–1226. 16731999 10.2337/dc05-2465

[17] Law MR, Wald NJ, Rudnicka AR (2003) Quantifying effect of statins on low density lipoprotein cholesterol, ischaemic heart disease, and stroke: systematic review and meta-analysis. BMJ 326: 1423. 12829554 10.1136/bmj.326.7404.1423 PMC162260

[18] Smith MEB, Lee NJ, Haney E, Carson S (2009) Drug class review: Hmg-coa reductase inhibitors (statins) and fixed-dose combination products containing a statin: Final report update 5. Portland (OR). 21089253

[19] Weng TC, Yang YH, Lin SJ, Tai SH (2010) A systematic review and meta-analysis on the therapeutic equivalence of statins. J Clin Pharm Ther 35: 139–151. 20456733 10.1111/j.1365-2710.2009.01085.x

[20] Jadad AR, Moore RA, Carroll D, Jenkinson C, Reynolds DJ, et al (1996) Assessing the quality of reports of randomized clinical trials: is blinding necessary? Control Clin Trials 17: 1–12. 8721797 10.1016/0197-2456(95)00134-4

[21] Cook RJ, Sackett D (1995) The number needed to treat: a clinically useful measure of treatment. BMJ 310: 452–454. 7873954 10.1136/bmj.310.6977.452 PMC2548824

[22] The Cochrane Collaboration (2011) Review Manager (RevMan) [Computer program]. Version 5.1. Copenhagen: The Nordic Cochrane Centre, The Cochrane Collaboration, 2011.

[23] Scholten RJ, Kostense PJ, Assendelften WJ, Bouter LM (1999) De praktijk van systematische reviews. IV. Het combineren van de resultaten van afzonderlijke onderzoeken. [In Dutch] Ned Tijdschr Geneeskd 143: 786–791. 10347641

[24] Higgins JP, Thompson SG, Deeks JJ, Altman DG (2003) Measuring inconsistency in meta-analysis. BMJ 327: 557–560. 12958120 10.1136/bmj.327.7414.557 PMC192859

[25] Pyrälä K, Pedersen TR, Kjekshus J, Faergeman O, Olsson AG, et al (1997) Cholesterol lowering with simvastatin improves prognosis of diabetic patients with coronary heart disease. A subgroup analysis of the Scandinavian Simvastatin Survival Study (4S). Diabetes Care 20: 614–620. 9096989 10.2337/diacare.20.4.614

[26] Goldberg RB, Mellies MJ, Sacks FM, Moyé LA, Howard BV, et al (1998) Cardiovascular events and their reduction with pravastatin in diabetic and glucose-intolerant myocardial infarction survivors with average cholesterol levels: subgroup analyses in the cholesterol and recurrent events (CARE) trial. The Care Investigators. Circulation 98: 2513–2519. 9843456 10.1161/01.cir.98.23.2513

[27] Collins R, Armitage J, Parish S, Sleigh P, Peto R (2003) MRC/BHF Heart Protection Study of cholesterol-lowering with simvastatin in 5963 people with diabetes: a randomised placebo-controlled trial. Lancet 361: 2005–2016. 12814710 10.1016/s0140-6736(03)13636-7

[28] Keech A, Colquhoun D, Best J, Kirby A, Simes LA, et al (2003) Secondary prevention of cardiovascular events with long-term pravastatin in patients with diabetes or impaired fasting glucose: results from the LIPID trial. Diabetes Care 26: 2713–2721. 14514569 10.2337/diacare.26.10.2713

[29] Post Coronary Artery Bypass Graft Trial Investigators (1997) The effect of aggressive lowering of low-density lipoprotein cholesterol levels and low-dose anticoagulation on obstructive changes in saphenous-vein coronary-artery bypass grafts. N Engl J Med 336: 153–162. 8992351 10.1056/NEJM199701163360301

[30] GISSI Prevenzione Investigators (Gruppo Italiano per lo Studio della Sopravvivenza nell'Infarto Miocardico) (2000) Results of the low-dose (20 mg) pravastatin GISSI Prevenzione trial in 4271 patients with recent myocardial infarction: do stopped trials contribute to overall knowledge? Ital Heart J 1: 810–820. 11302109

[31] Serruys PW, de Feyter R, Macaya C, Kokott N, Puel J, et al (2002) Fluvastatin for prevention of cardiac events following succesfull first percutaneous coronary intervention: a randomized controlled trial. JAMA 287: 3215–3222. 12076217 10.1001/jama.287.24.3215

[32] Cardiovasculair risicomanagement (2012) (Tweede herziening) [In Dutch] Huisarts Wet 55. (1): 14–28.

[33] Perk J, De Backer G, Gohlke H, Graham I, Reiner Z, et al (2012) European Guidelines on cardiovascular disease prevention in clinical practice (version 2012). The Fifth Joint Task Force of the European Society of Cardiology and Other Societies on Cardiovascular Disease Prevention in Clinical Practice (constituted by representatives of nine societies and by invited experts). Eur Heart J 33: 1635–1701. 22555213 10.1093/eurheartj/ehs092

[34] Stark Casagrande S, Fradkin JE, Saydah SH, Rust KF, Cowie CC (2013) The prevalence of meeting A1C, blood pressure, and LDL goals among people with diabetes, 1988–2010. Diabetes Care 36: 227–229. 10.2337/dc12-2258 PMC3714503 23418368

[35] Stone NJ, Robinson J, Lichtenstein AH, Bairey Merz CN, Blum CB, et al. (2013) ACC/AHA guideline on the treatment of blood cholesterol to reduce atherosclerotic cardiovascular risk in adults: a report of the American College of Cardiology/American Heart Association Task Force on Practice Guidelines. Circulation; 129 [suppl 2]: S1–S45. 10.1161/01.cir.0000437738.63853.7a 24222016

[36] Armitage J (2007) The safety of statins in clinical practice. Lancet 370: 1781–1790. 17559928 10.1016/S0140-6736(07)60716-8

[37] Silva M, Matthews ML, Jarvis C, Nolan NM, Belliveau P, et al (2007) Meta-analysis of drug-induced adverse events associated with intensive-dose statin therapy. Clin Ther 29: 253–260. 17472818 10.1016/j.clinthera.2007.02.008

[38] Newman C, Tsai J, Szarek M, Luo D, Gibson E (2006) Comparative safety of atorvastatin 80 mg versus 10 mg derived from analysis of 49 completed trials in 14,236 patients. Am J Cardiol 97: 61–67. 16377285 10.1016/j.amjcard.2005.07.108

[39] Ko DT, Wijeysundera HC, Jackevicius CA, Yousef A, Wang J, et al (2013) Diabetes mellitus and cardiovascular events in older patients with myocardial infarction prescribed intensive-dose and moderate-dose statins. Circ Cardiovasc Qual Outcomes 6: 315–322. 23674307 10.1161/CIRCOUTCOMES.111.000015

[40] Fintel D, Joyce A, Mackell J, Graff J, Kuntze E, et al (2007) Reduced mortality rates after intensive statin therapy in managed-care patients. Value Health 10: 161–169. 17391425 10.1111/j.1524-4733.2006.00163.x

